# Dynamics of Silurian Plants as Response to Climate Changes

**DOI:** 10.3390/life11090906

**Published:** 2021-08-31

**Authors:** Josef Pšenička, Jiří Bek, Jiří Frýda, Viktor Žárský, Monika Uhlířová, Petr Štorch

**Affiliations:** 1 Centre of Palaeobiodiversity, West Bohemian Museum in Pilsen, Kopeckého sady 2, 301 00 Plzeň, Czech Republic; muhlirova@zcm.cz; 2 Laboratory of Palaeobiology and Palaeoecology, Geological Institute of the Academy of Sciences of the Czech Republic, Rozvojová 269, 165 00 Prague 6, Czech Republic; bek@gli.cas.cz (J.B.); viktor.zarsky@natur.cuni.cz (V.Ž.); Storch@gli.cas.cz (P.Š.); 3 Faculty of Environmental Sciences, Czech University of Life Sciences Prague, Kamýcká 129, 165 21 Praha 6, Czech Republic; bellerophon@seznam.cz; 4 Czech Geological Survey, Klárov 3/131, 118 21 Prague 1, Czech Republic; 5 Department of Experimental Plant Biology, Faculty of Science, Charles University, Viničná 5, 128 43 Prague 2, Czech Republic; 6 Institute of Experimental Botany of the Czech Academy of Sciences, v. v. i., Rozvojová 263, 165 00 Prague 6, Czech Republic; 7 Institute of Geology and Palaeontology, Faculty of Science, Charles University, Albertov 6, 128 43 Prague 2, Czech Republic

**Keywords:** early land plants, Silurian, plant assemblages, palaeoclimatic changes, polysporangiate plants, plant stress responses, carbon cycle

## Abstract

The most ancient macroscopic plants fossils are Early Silurian cooksonioid sporophytes from the volcanic islands of the peri-Gondwanan palaeoregion (the Barrandian area, Prague Basin, Czech Republic). However, available palynological, phylogenetic and geological evidence indicates that the history of plant terrestrialization is much longer and it is recently accepted that land floras, producing different types of spores, already were established in the Ordovician Period. Here we attempt to correlate Silurian floral development with environmental dynamics based on our data from the Prague Basin, but also to compile known data on a global scale. Spore-assemblage analysis clearly indicates a significant and almost exponential expansion of trilete-spore producing plants starting during the Wenlock Epoch, while cryptospore-producers, which dominated until the Telychian Age, were evolutionarily stagnate. Interestingly cryptospore vs. trilete-spore producers seem to react differentially to Silurian glaciations—trilete-spore producing plants react more sensitively to glacial cooling, showing a reduction in species numbers. Both our own and compiled data indicate highly terrestrialized, advanced Silurian land-plant assemblage/flora types with obviously great ability to resist different dry-land stress conditions. As previously suggested some authors, they seem to evolve on different palaeo continents into quite disjunct specific plant assemblages, certainly reflecting the different geological, geographical and climatic conditions to which they were subject.

## 1. Introduction

The final stages of the colonization of land by plants (Embryophyta) within the Ordovician-Silurian interval is one of the most important global events in the evolution of life on the Earth. Wellman and Strother [1] and Kenrick [2] hypothesized that life existed on the land since over 2.7 Gyr in the form of communities of bacteria (including cyanobacteria) and archaea [3,4], that colonized shallow places in the fresh-water environment. 

Successful terrestrialization of plants was enabled by some crucial adaptations including tissues ensuring the distribution of water and nutrients for growth, body protection against desiccation and UV, anchoring in the substrate, and propagation by resistant spores [5,6].

Ordovician and Silurian plant communities already included possibly several groups of polysporangiate land plants (no monosporangiates are documented by the fossil record), including more ancient and enigmatic cryptosporophytes [7,8], accompanied by bacterial-cyanobacterial mats [9,10,11]. At the end of the Silurian and the beginning of the Devonian it is already possible to quite reliably identify the first lycophytes (*Baragwanathia* and *Asteroxylon*).

The first evidence indicating the existence of land plants are cryptospores of the Early Middle Ordovician age (c. 470MA) [12]. However, their producers are still unknown. Some very questionable polysporangiophytes have been described from the Ordovician [13,14] that coincide with the appearance of the first true trilete spores within the Late Ordovician [15,16]. These spores indicate an earlier origin of possibly several types of polysporangiophyte-grade plants, probably during Ordovician. Some authors hypothesized the colonization of land by embryophytes during the Cambrian (or even Proterozoic) and this recently has been strongly supported by phylostratigraphic data [3,4,17,18].

**Figure 1:**
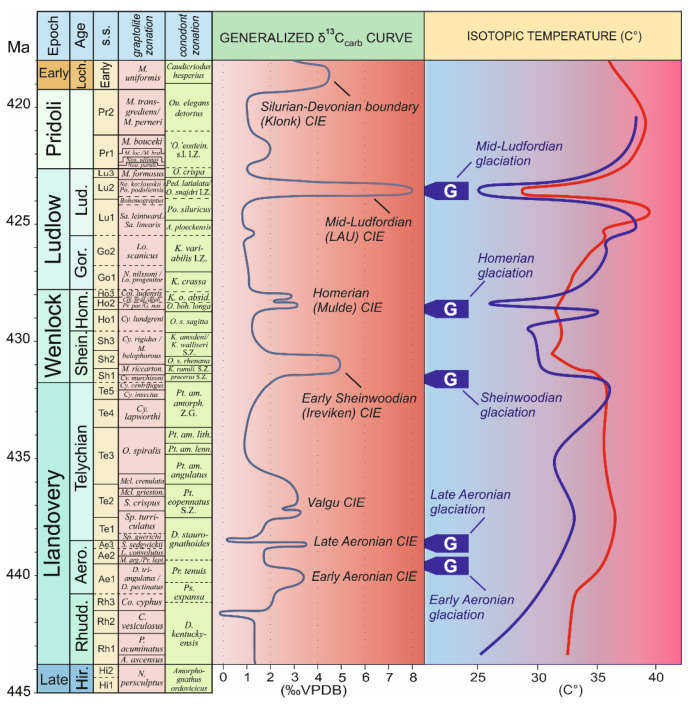
Silurian time scale showing conodont and graptolite biozones, stage slices (s.s.), and generalized δ^13^Ccarb curve, modified from [20,21,22], combined with isotopic temperature curves (blue based on brachiopod, and red on conodont δ^18^O data) from [23]. The conodont isotopic temperature curves for Ludfordian were modified based on [24]. The isotopic temperatures assume “ice-free” conditions (δ^18^Oseawater = −1.1‰ vs. VSMOW). Abreviations: Shein.—Sheinwoodian; Hom.—Homerian; Gor.—Gorstian; Lud.—Ludfordian; Loch.—Lochkovian. Abreviations used in graptolite column, bottom to top: *Cy.—Cyrtograptus; M. riccarton.—Monograptus riccartonensis; Pr. par./G. nas.—Pristiograptus parvus/Gothograptus nassa; Col.—Colonograptus; Col. prae./deub.—Col. praedeubeli/Col. deubeli; N./ Lo.—Neodiversograptus/ Lobograptus; Lo.—Lobograptus; Sa. leintward./Sa. Linearis—Saetograptus linearis; Ne./Po.—Neocucullograptus/Polonograptus; Neo.—Skalograptus; Neo. parult.—Neocolonograptus parultimus; M. loc./M. bra.—Skalograptus lochkovensis/M. lochkovensis branikensis; M.—Monograptus. Conodont column, bottom to top: procerus—Pterospathodus pennatus procerus; K.—Kockelella; K. ranuli.—K. ranuliformis; O. s. rhenana—Ozarkodina sagitta rhenana; O. s. sagitta—Ozarkodina sagitta sagitta; O. boh. Longa—Ozarkodina bohemica longa; K. o. absid.—Kockelella ortus absidata; A.—Ancoradella; Po.—Polygnathoides; Ped.—Pedavis; O.—Ozarkodina; ‘O.’ eostein.—“Ozarkodina” eosteinhornensis sensu lato; Ou.—Oulodus*. S.Z.—Superbiozone; Z.G.—Zonal Group; I.Z.—Interval Biozone.

This paper provides an overview and a revision of Silurian plant assemblages (from Wenlock to Přídolí) of polysporangiophytes with dispersed spores and cryptospores. Special attention is paid to the dynamics of the Silurian plant evolution within the context of Silurian climate changes linked with perturbations of the global carbon cycle (Figure 1). The data on Llandoverian plants are not included in the latter analysis because (1) observations of well-preserved macroplants below the Wenlock are extremely rare and (2) the plants are represented only by sterile bifurcated axes [19].

### 1.1. Character of Early Land Plants and Their Classification

Gerrienne et al. [25] established a currently widely accepted approach for the classification of the earliest embryophytes (including monosporangiate hornworts and mosses not reported from fossil data), which, along with obscure embryophytes and probably bacterial-cyanobacterial mats, formed essential components of the entire terrestrial ecosystem, including the well-known genera *Nematothallus*, *Pachytheca* and *Prototaxites*. *Nematothallus,* which probably belonged to lichenized fungi [26]. Many different-sized tubular structures from Silurian-Devonian sediments (probably composed of hyphae) are assigned to *Prototaxites*. *Pachytheca*, represented by small spherical structures of still unknown affinity, stressed some similarities to *Prototaxites* [27]. *Nematasketum*, classified as an enigmatic non-embryophyte plant [28], was described based on vegetative parts and possesses an axial organization with peripheral tissues forming a ring. *Nematasketum* shows some similarities with *Prototaxites* [28].

The first credible fossil bryophytes, represented by *Riccardiothallus devonicus* [15,29], are of the Pragian (Early Devonian) age. Morris et al. [15] estimated the appearance of bryophytes during the Late Cambrian–Late Ordovician interval based on molecular clock data. The Ordovician occurrence of bryophytes would also be supported by fossil spore exine ultrastructural similar to some spores of recent liverworts [30]. However, there is a recent consensus of monophyletic origin of the bryophytes and its sister relationship with tracheophytes (i.e., tracheophytes did not evolve from bryophytes [18,31]). This clearly indicates that it might not make much sense to speak about monosporangiate mosses in the Ordovician or Silurian without fossil support for a polysporangiate common ancestor of both mosses and tracheophytes, which is currently probable (see our Discussion). 

Polysporangiophytes [25,32,33] formed the only embryophytic plants documented by the fossil in the Silurian. The group includes the first true vascular plants (Tracheophyta). Although recent vascular plants may reach tens of meters in height, the earliest terrestrial forms were mostly diminutive [8,34,35,36,37], although some forms of polysporangiophytes could already have been over 10 cm tall (e.g., *Tichavekia grandis*) [38]. The main character of polysporangiophytes is, as the name suggests, the branched sporophyte with more than one sporangium [39]. Silurian plant communities most commonly include polysporangiophyte taxa, such as *Cooksonia*, *Salopella*, *Aberlemnia*, *Hedeia*, *Tortillicaulis*, *Fusiformitheca*, *Steganotheca* and *Caia.* Sterile fragments of plant axes are referred to the genus *Hostinella*. All those genera are usually placed among “rhyniophytoids“ [40].

Basal lycophytes are represented by zosterophylls [33]. Silurian zosterophylls include *Zosterophyllum*, *Bathurstia*, *Distichophytum*, *Macivera*, and *Aberlemnia* with bivalved sporangia and dehiscence along the distal sporangial margin, which has led to the placement of these plants [38,41] in the lycophyte stem group. The first unambiguous lycophyte is *Baragwanathia*, occurring in the late Silurian [42,43,44], which already had well-developed microphylls.

### 1.2. Silurian Plant Assemblages

Silurian plant assemblages and their geographical distribution were studied in several papers [45,46,47,48,49]. We must be aware that only a limited number of plants can be found at one locality and it is not exceptional that only one species of polysporangiophyte occurs at a locality. It appears that phytogeographical differentiation and disjunction (isolation) of land-plant types/groups was significant during the Late Silurian (Přídolí) and Early Devonian [47,50]. Raymond et al. [47] established 35 macrofloristic assemblages for Silurian to Early Devonian times based on correspondence and cluster analyses for different geographical areas and climatic zones. The latter analyses resulted in the establishment of four phytogeographic units: (1) North Laurussian unit (Bathurst Island) located near the palaeoequator; (2) South Laurussian–Northwest Gondwanan unit (UK; Podolia, Ukraine and Bolivia) located approximately 18° S palaeolatitude to 60–75° S palaeolatitude; (3) Kazakhstanian unit located north of the palaeoequator; and (4) Northeast Gondwanan (Australian) unit at 10° S palaeolatitude [47]. Later Wellman et al. [48] established 14 assemblages but they did not revise palaeophytogeographical areas proposed by Raymond et al. [47] that are used herein.

## 2. Materials and Methods

Specimens/samples were selected from the Collections of the Centre of Palaeobiodiversity of the West Bohemian Museum in Pilsen—WBM (Czech Republic), specimens F21761a, F21762; SEM-rack SILI-A1, Kosov Quarry; National Museum—NM (Prague, Czech Republic), specimens D-475 Kosov Quarry, D-552b Loděnice, Špičatý vrch-Barrandovy Jámy; Czech Geological Survey (Prague, Czech Republic)—CGS, specimen KR 1, Karlštejn locality; Faculty of Sciences, Charles University, Prague (Czech Republic)—FSCU, Kosov Quarry; Swedish Museum of Natural History (Stockholm, Sweden)—SMNH, specimen JE-Sch0260B, Kosov Quarry; private collections of Ondřej Zicha—PCC (Czech Republic), Špičatý vrch-Barrandovy Jámy and the collection of the University of Saskatchewan—US (Saskatoon, Canada), specimens US600-6791, US600-8144, US384-8137, US688-8152, US385-2398, Bathurst Island, Canada. Specimens from NM, WBM, CGS, FSCU, SMNH and PCC represented polysporangiophyte assemblages from the temperate zone of the lower Wenlock (Sheinwoodian) and Přídolí of the Barrandian area, the Prague Basin (Czech Republic). Mesofossils from the upper Wenlock (Homerian, *parvus-nassa* Biozone) of the Lištice locality are stored in WBM showing small plant fragments macerated from the rock (HCL 32% (5–10 days)-HF35% (5–8 days)-HNO3 65% (several minutes–10 days). Specimens of polysporangiophytes from the tropical zone of the Gorstian age, Ludlow (Canada) are stored in the US.

## 3. Results

### 3.1. Environment of Silurian Plants

Early land/polysporangiate plants diversified and colonized continents during Silurian-Devonian “terrestrial radiation” [51]. Recent analysis convincingly indicates a possibility that a great land plant radiation had already occurred in the Ordovician and argues that land plants were possibly not affected by the end-Ordovician extinction event [52]. Servais et al. [53] stated the importance of environmental factors, including atmospheric composition and the geochemical carbon cycle [54,55], as major influencers of the evolutionary dynamics of early plants. The Silurian Period has been considered as a stable interval characterized by gradual, long-term climatic change starting from the Middle to Late Ordovician cold climate and culminating in Silurian greenhouse conditions [56,57]. The Silurian, lasting from approximately 443.1 to 419.0 million years [22], represents a key interval in the biological evolution of marine ecosystems [58,59,60,61]. The transitional character of the Silurian is not illustrated only by the changes of marine communities and ecosystems, but also by the instability of the global carbon cycle including rapid changes in atmospheric pO_2_ and pCO_2_ [62,63,64]. Published data indicate that during the relatively short Silurian interval at least five globally observed carbon isotope excursions have been recognized (Figure 1). These anomalies include the δ^13^C excursions in the mid-Llandovery, early Wenlock, late Wenlock, late Ludlow, and across the Silurian-Devonian boundary [21,65]. Geochemical events were closely linked to major crises in marine ecosystems as well as to palaeoclimatic changes [24,56,66,67,68,69].

Global temperature, together with total precipitation, are key parameters influencing terrestrial community development. However, the reconstruction of global temperature change is complicated and has to be based on samples from different palaeoregions because ocean temperature varied with season, water depth, and palaeolatitude [23]. In addition, to calculate palaeotemperatures from marine calcite or apatite δ^18^O values, the oxygen isotope composition of the Silurian seawater has to be assumed. However, there is no consensus on whether the oxygen isotope composition of seawater evolved during the Phanerozoic [70,71] or was more or less constant [72]. Thus, without a better constrained Silurian seawater δ^18^O, the calculation of absolute temperatures remains speculative. However, relative temperature changes can be calculated independent of seawater δ^18^O (Figure 1).

Grossman and Joachimski [23] mentioned that the oxygen isotope record for Silurian brachiopods and conodonts from tropical and subtropical palaeolatitudes showed a Llandovery Warm Trend. On the other hand, Silurian brachiopod data revealed a Ludfordian Cool Event. New δ^18^O conodont data from Ludfordian temperate as well as tropical palaeolatitudes [24] confirms the marked cooling of sea surface temperatures at time a significant eustatic sea-level fall occurred on several palaeocontinents. These observations, interpreted as a major glaciation in polar and subpolar Gondwana, are referred to as the ‘Mid-Ludfordian Glaciation’ [24].

Taken together, recently available sedimentological (glacial diamictite deposition) and geochemical (δ^18^O) data suggest at least five glacial events within the Silurian Period, namely early Aeronian, late Aeronian, early Sheinwoodian, Homerian, and mid-Ludfordian glaciation [24,56,66,69,73] (Figure 1).

### 3.2. Diversity of Silurian Plants 

Raymond et al. [47] proposed the following palaeophytogeographical units.

#### 3.2.1. North Laurussian Unit

**Figure 2:**
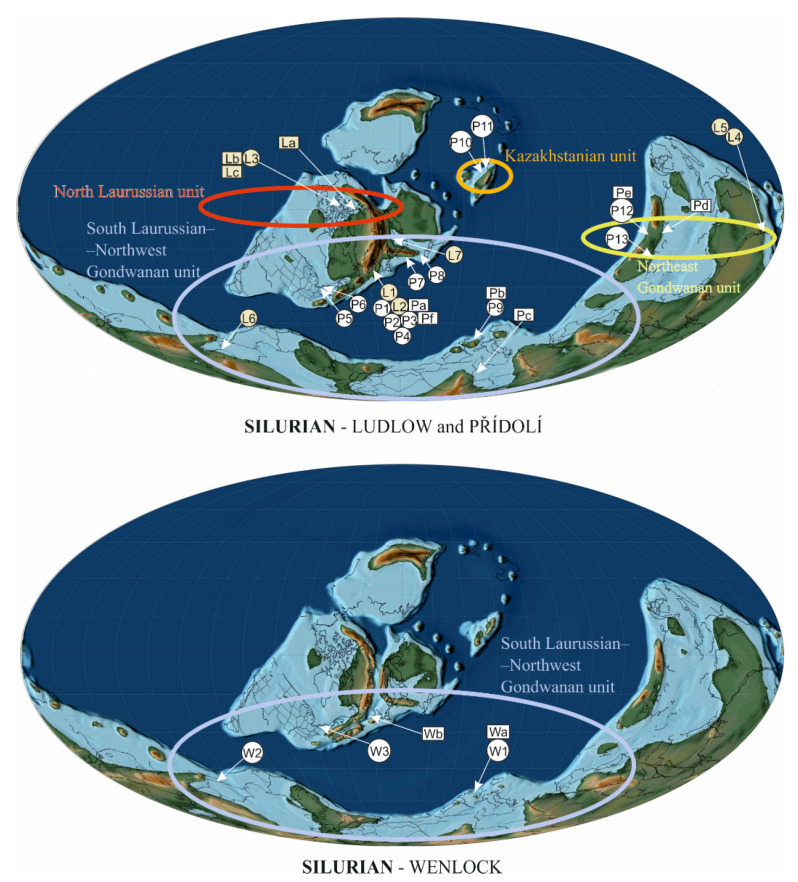
Silurian palaeocontinental reconstructions according to Scotese [76] plotting palaeogeographical distribution of plants (rectangle) or plant assemblages (circle). Colored ellipses represent individual phytogeographic units according to Raymond et al. [48]; W = Wenlock, L = Ludlow, P = Přídolí.

**Figure 3:**
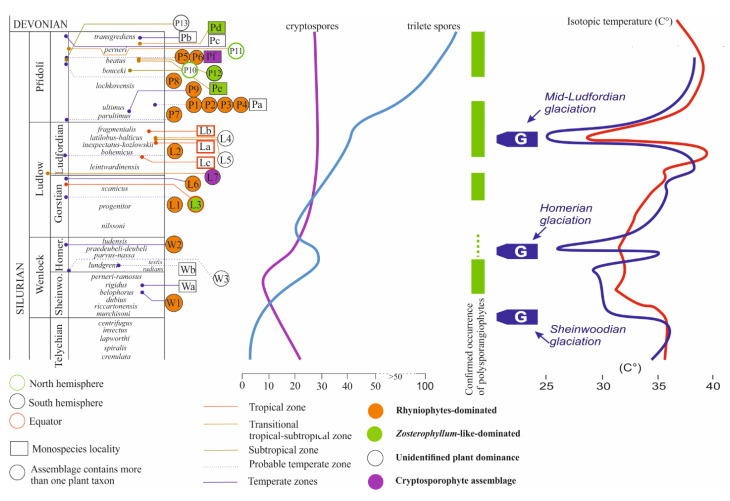
Stratigraphical distribution of plants or plant assemblages from the Wenlock (W) through Ludlow (L) to Přídolí (P). Quantitative curves of cryptospores (see Figure 8) and isotopic temperature curves (see Figure 1) put together. The circle at the end of the connecting line between the assemblage/plant unit and stratigraphy levels indicates the accuracy of the stratigraphic determination-the more right this point is, the more accurate the determination. **Assemblages**: W1 = Barrandian area, the Prague Basin, Czechia [77,78,79,80]; W2 = Souther Bolivia [89]; W3 = Gasport Stone Quarry, Niagara County, New York State, USA [90]; L1 = Welsh Basin (Cwm Craig Ddu, Powys) [91]; L2 = Welsh Basin (Capel Horeb, Powys) [90]; L3 = Bathurst Island, Arctic Canada [74]; L4 = Victoria, Australia, Centennial Beds [42,92]; L5 = Victoria, Australia, *Monograptus* Beds [42,92]; L6 = Tarija, southern Bolivia [93]; L7 = Gotland, Sweden [94]; P1 = Anglo-Welsh Basin (Ludford Corner, Ludlow) [95]; P2 = Anglo-Welsh Basin (Perton Lane, Hereford) [96,97,98]; P3 = Anglo-Welsh Basin (Freshwater East, Pembrokeshire) [99]; P4 = Anglo-Welsh Basin (Capel Horeb, Powys) [91]; P5 = New York State, USA [90]; P6 = Ontario, Canada [90]; P7 = Holy Cross Mts., Poland [100]; P8 = Podolia, Ukraine [101]; P9 = Barrandian area, the Prague Basin, Czechia [38,43]; P10 = Kazakhstan [45]; P11 = Kazakhstan, Balkhash area [102,103]; P12 = Junggar Basin, Xinjiang, China [104]; P13 = Northern Vietnam [105]; **Isolated finding of plants**: Wa = Barrandian area, the Prague Basin, Czechia [83]; Wb = Tipperary, Ireland [106]; La = Northern Greenland [75]; Lb = Bathurst Island, Arctic Canada [74]; Lc = Bathurst Island, Arctic Canada [74]; Pa = Anglo-Welsh Basin (Little Wallop Hall, Shropshire) [107]; Pb = Barrandien area, the Prague Basin, Czechia [82]; Pc = Homra Basin, Libya [108]; Pd = Yunan, China [50]; Pe = Junggar Basin, Xinjiang, China [104]; Pf = Shropshire, UK [7].

**Figure 4:**
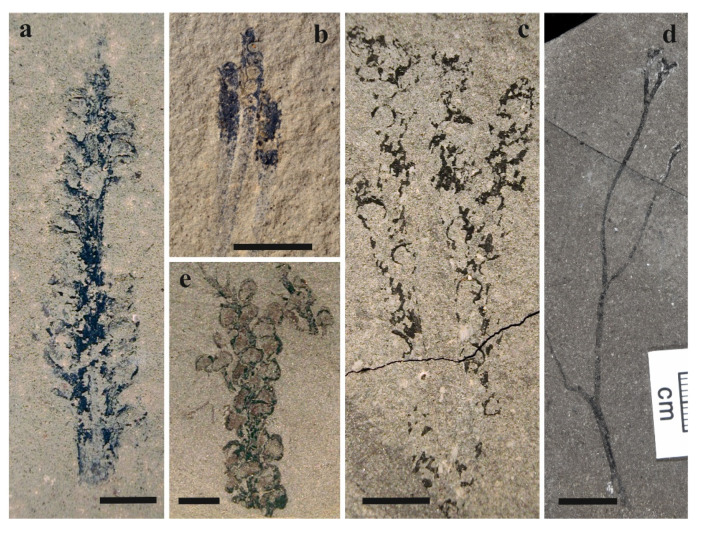
Tropical flora assemblage from Bathurst Island, Canada. (**a**) cf. *Bathurstia*, US—US600-6791, scale bar = 5 mm; (**b**) *Distichophytum* sp. US—US600-8144, scale bar = 10 mm; (**c**) aff. *Zosterophyllum* sp. A, US—US688-8152, scale bar = 5 mm; (**d**) *Macivera gracilis*, US—US385-2398, scale bar = 10 mm; (**e**) *Zosterophyllum* sp., US—US384-8137, scale bar = 5 mm. Used with the permission of the authors [74].

This unit, located near the palaeoequator, was of tropical character (Figure 2—La, Lb, Lc, L3, La) and includes north Laurussian basins. The oldest plant fossil record is known from the Ludlow strata (Figure 3) of Bathurst Island [45,74] and North Greenland [75]. The Bathurst Island plant assemblage provides several terrestrial species, e.g.,: Cf. *Bathurstia* sp., *Zosterophyllum* sp., *Distichophytum* sp., Aff. *Zosterophyllum* sp. A, *Macivera gracilis* [74] (Figure 4). The only plant from North Greenland is *Salopella* sp. [75]. Generally, this plant assemblage can be characterized as a zosterophyll-dominated flora with minor rhyniophytes.

#### 3.2.2. South Laurussian–Northwest Gondwanan Unit

This unit includes territories from approximately 18° S palaeolatitude to 60–75° S palaeolatitude [75] and was probably a part of the temperate climatic zone (Figure 2). The fossil record of this palaeophytogeographical unit ranges from early Wenlock (Sheinwoodian) to Přídolí.

##### Northwest Gondwana

**Figure 5:**
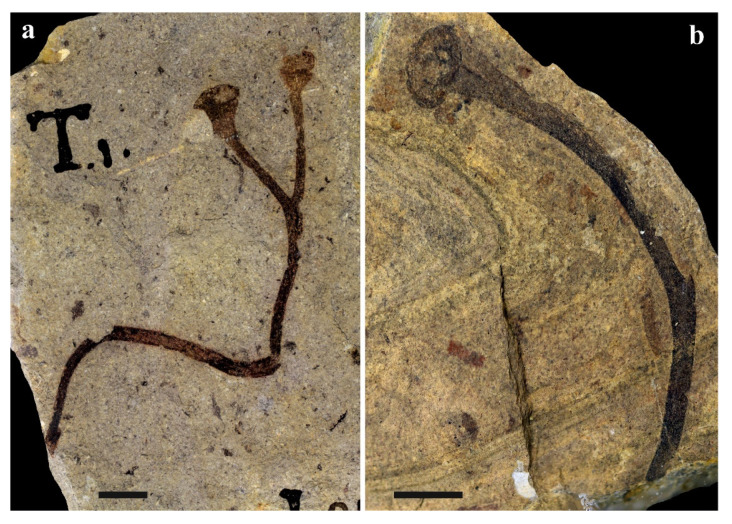
Plant fossils from the South Laurussian–Northwest Gondwanan unit (temperate zone), Wenlock, Scheinwoodian, Barrandian area (the Prague Basin), Czechia; (**a**) *Cooksonia barrandei*, specimen D-552b, NM, Špičatý vrch-Barrandovy Jámy; (**b**) *Cooksonia* sp. PCC (notice—will be a part of collection of NM), Špičatý vrch-Barrandovy Jámy; scale bar for both specimens = 5 mm.

**Figure 6:**
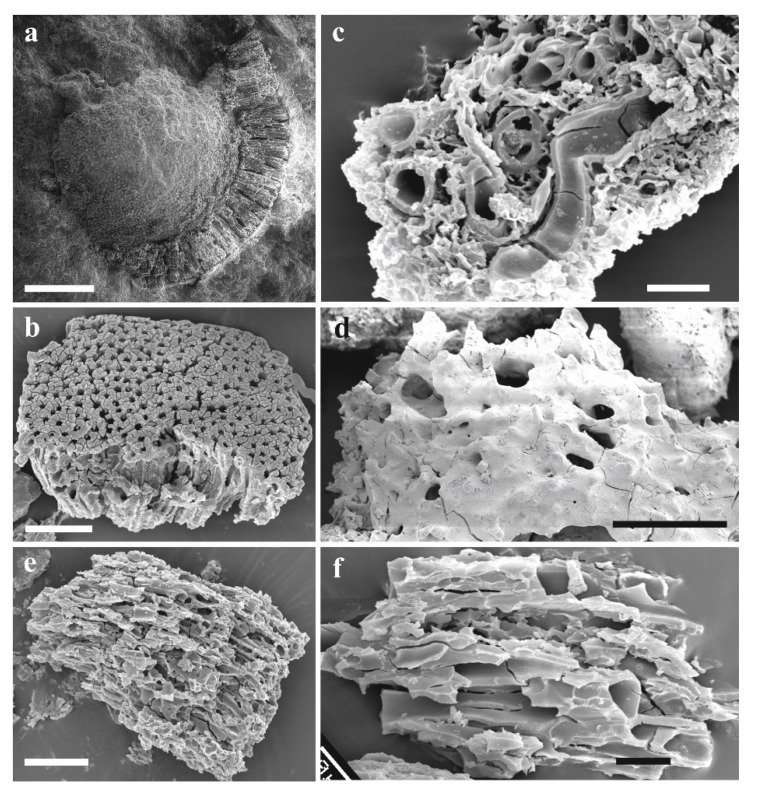
Plant assemblage from the South Laurussian–Northwest Gondwanan unit (temperate zone) known from the Homerian of the Barrandian area, the Prague Basin (*parvus-nassa* Biozone); (**a**) *Pachytheca* showing differentiated medulla (inner) and cortex (outer par), scale bar = 500 µm; (**b**) Cortical fragment of *Pachytheca* in detail composed of densely spaced radial tubes, scale bar = 20 µm; (**c**) Non-embryophyte plant cf. *Nematasketum* showing unevenly thickened tubes, SEM-rack SILI-A1, scale bar = 200 µm; (**d**) Cf. *Cosmochlaina* showing outer covering layer being partly perforated, scale bar = 200 µm; (**e**) Varying diameter of tubes of *Prototaxites*, scale bar = 100 µm; (**f**) Detail of tubes of *Prototaxites*, scale bar = 20 µm. All specimens were observed under SEM.

Northwest Gondwana (peri-Gondwana) provides the oldest true vascular land plants, *Cooksonia barrandei* and *Cooksonia* sp. (Figure 5), from the Czech Republic (Barrandian area, the Prague Basin) [77,78]. These globally important specimens come from the *Monograptus belophorus* Biozone of the Sheinwoodian Age. Sheinwoodian localities of the Barrandian area yielded several unpublished early land plants of different affinities (rhyniophytes, probably zosterophylls, cryptosporophytes) [77,78,79,80]. Current research suggests that cooksonioid dominated plant assemblages were accompanied by Ascomycota [81], *Prototaxites* sp., and enigmatic *Pachytheca* [82]. Libertín et al. [83] also recorded *Cooksonia* sp. from the same geographic area but from the stratigraphically younger *Cyrtograptus rigidus* Biozone (Figure 3—Wa). Generally, the latter assemblage (Figure 3—W1 plus Wa) is characterized by relatively rich vegetation cover of the temperate zone of the Southern hemisphere. The other probable plants, such as *Prototaxites* sp. (Figure 6e,f), *Pachytheca* sp. (Figure 6a,b), cf. *Cosmochlaina* (Figure 6d), and cf. *Nematasketum* (Figure 6c) belong to relatively abundant fossils in the Homerian interval. 

**Figure 7:**
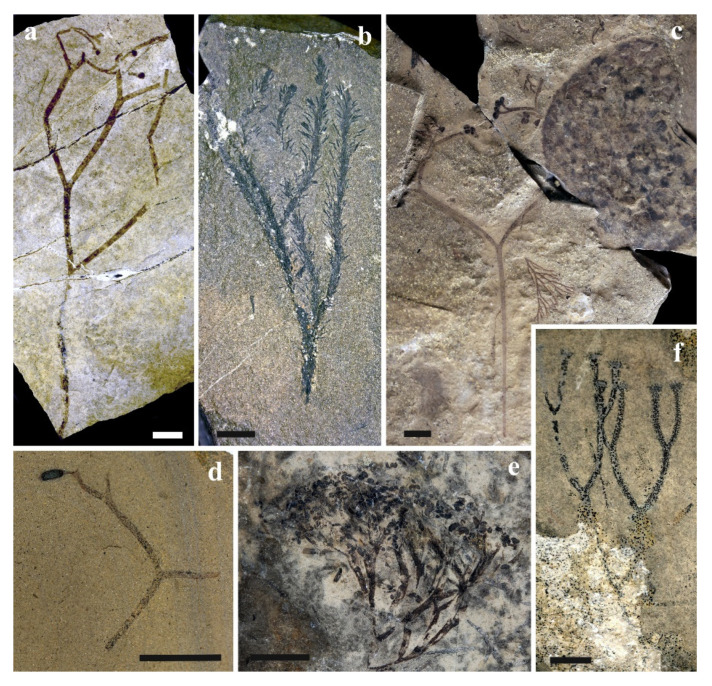
Plant assemblage from the South Laurussian–Northwest Gondwanan unit (temperate zone) from Přídolí (*S. parultimus-S. ultimus* Biozone) of the Barrandian area, the Prague Basin, Czechia; (**a**) *Cooksonia* cf. *hemisphaerica*, specimen of NM—D-475, Kosov Quarry; (**b**) *Baragwanathia brevifolia*, specimen of CGS—KR 1, Karlštejn locality; (**c**) *Tichavekia grandis*, specimen of WBM—F21761a, Kosov Quarry; (**d**) Cf. *Fusiformitheca* sp., specimen of WBM—F21762, Kosov Quarry; (**e**) *Aberlemnia bohemica*, specimen of SMNH—JE-Sch0260B, Dlouhá Hora locality; (**f**) *Cooksonia* sp., specimen of FSCU, Kosov Quarry. All scales 10 mm.

The relatively rich plant assemblage from the Barrandian area (the Prague Basin) comes from Přídolí strata (Figure 2—P9, Pb). Mineral nutrient-rich volcanic islands formed from late Wenlock–early Ludlow Kosov and Svatý Jan volcanic centres could have provided suitable conditions for terrestrialization of early land plants [38,82,84,85]. Plants from the *Skalograptus parultimus-Skalograptus ultimus* Biozone interval (Figure 3—P9) include *Cooksonia* sp. (Figure 7f), *Cooksonia* cf. *hemisphaerica* (Figure 7a), *Aberlemnia bohemica* (Figure 7e), *Fusiformitheca* sp. (Figure 7d), and *Tichavekia grandis* (Figure 7c) [38,86,87]. Not only fertile but also abundant sterile axial parts of *Cooksonia* affinity are reported [82,88]. Occurrence of the basal lycophyte *Baragwanathia brevifolia* (Figure 7b) [43] from *Skalograptus ultimus* Biozone is unique, representing the single finding of a lycophyte with differentiated microphylls standing out among other Silurian plants of the Barrandian area, which, in contrast, exhibit ancient rhyniophytoid characters. 

Sterile rhyniophytes were described from the Late Wenlock/Ludlow strata of southern Bolivia [89] (Figure 3—W2). Stratigraphically younger specimens from the Lipeón Formation at Tarija of Ludlow age yielded cf. *Aberlemnia caledonica*, sterile axes assigned to the genus *Hostinella*, and isolated sporangia determined based on their morphology as cf. *Cooksonia hemisphaerica,* cf. *Tarrantia* and isolated sporangia [93] (Figure 3—L6). One of the best-preserved specimens of cf. *Aberlemnia caledonica* was earlier published by Morel et al. [109].

Daber [108] described the uppermost Přídolí flora from Libya (Figure 2—Pc), including *Cooksonia* sp. This area was situated near the northern edge of the Gondwana mainland, far south of the Barrandian area (the Prague Basin) during Přídolí times.

##### South Laurussia

The South Laurussian unit includes basins located in southwest Laurussia, situated approximately 30° south latitude [76], and belonging to temperate zone (Figure 2). One of those basins, the classic Welsh Basin, UK, has a sedimentological record ranging from Cambrian to Early Devonian. Rhyniophytoids are the only plants reported from the Late Silurian and Early Devonian of this area. The oldest polysporangiophyte, *Cooksonia* sp. (Figure 3—Wb) from Homerian strata of the Tipperary County (Ireland) of Laurussia, was described by Edwards et al. [106]. This area was located probably c. 25° S palaeolatitude [76]. It is not possible to speculate about the general character of this vegetation because only one species has been recorded.

Gorstian assemblages from the Welsh-Basin probably belonging to the same phytogeographical unit are known from the Anglo-Welsh Basin, UK [91] (Figure 2—L1). They are typified by rhyniophytoid-dominated vegetation with *Cooksonia* sp., cf. *Cooksonia cambrensis*, *Cooksonia pertoni* and sterile axes of *Hostinella* (Figure 3—L1). 

Freshwater East in Pembrokeshire is a famous locality in the Welsh Basin (Figure 3—P3) that yielded a rich plant assemblage of the Přídolí age. *Cooksonia* and sterile axes of *Hostinella* were described by Edwards [99], including a species, *Cooksonia cambrensis*, and the rhyniophytoid *Tortilicaulis transwalliensis*, resembling sporophytes of some recent liverworts. 

A new rhyniophytoid-like species, *Caia langii,* was described from Perton Lane, UK [96] (Figure 3—P2), which is the original locality for *Cooksonia pertoni* [110]. Another plant from the same locality, *Pertonella dativelethra,* is characterized by distinctive sporangial projections [97]. The other plants from this site are *Cooksonia cambrensis* and *Salopella* sp. [98].

Ludford Corner, UK yielded exceptionally preserved plants (Figure 3—P1). Capel Horeb in Wales, UK (Figure 3—P4) is another locality of the Welsh Basin typified by coalified plant adpressions [91], such as *Steganotheca* and sterile axes of *Hostinella*. Rogerson et al. [107] described *Cooksonia pertoni* from Little Wallop Hall in Shropshire, UK (Figure 3—Pa). Similar flora is known from Ontario, Canada (Figure 3—P6) and New York, USA (Figure 3—P5). The polysporangiophytes from Canada and New York as coalified adpressions are preserved in fine-grained dolomite [90]. This area was located at the southeastern edge of the Laurussia palaeocontinent, approximately 40° south latitude [76] during Přídolí. The research was concentrated on six localities, of which *Cooksonia* sp., *Cooksonia* cf. *hemisphaerica*, and sterile axes assigned to *Hostinella* have been recorded.

Poland (eastern Laurussia) belonged to the South Laurussian–Northwest Gondwanan unit (Figure 3—P7) at approximately 20° south latitude [76]. Bodzioch et al. [100] published specimens from early Přídolí and assigned them to rhyniophytoids. Specimens come from the Holy Cross Mountains (Poland), including fertile *Cooksonia* sp. and sterile axes described as *Hostinella*.

Ishchenko [101] described an assemblage from the Přídolí dominated by rhyniophytoids including *Cooksonia pertoni* and *C. hemisphaerica* from Podolia (Ukraine). This area is typical for warm zones from the same latitude such as the Polish localities [100]. Unlike Poland *Zosterophyllum* sp., *Lycopodolica*, and *Eorhynia* (*Salopella*) are reported.

Wenlockian sterile branched axes were reported from the Gasport Stone Quarry, Niagara County, (New York, USA) [90] (Figure 2—W3). The section belongs to the graptolite zone of *Medusaegraptus*—a graptolite taxon that is also recognized in the upper Sheinwoodian to lower Homerian of the Barrandian region of the Prague Basin (Figure 3—W3). 

Edwards and Rogerson [91] published the Ludfordian rhyniophytoid-dominated vegetation from Capel Horeb of the Welsh Basin, UK (Figure 3—L2), including *Cooksonia* sp. and *Steganotheca striata*.

#### 3.2.3. Kazakhstanian Unit

This unit was located north of the palaeoequator (Figure 1) and its vegetation belongs to the subtropical zone. Two sites yielded plants, as seen on Figure 2 and Figure 3—P10, P11, and correspond with the stratigraphical interval of the *Skalograptus boucek –Skalograptus transgrediens* graptolite biozones. The Kazakhstanian unit was situated on the northern hemisphere in the subequatorial zone of the Palaeo-Tethys Ocean [76]. The assemblage includes the genera previously reported by Edwards and Wellman [45]. Other specimens described from the Balcan area are *Cooksonella sphaerica*, *Baragwanathia* sp., *Taeniocrada* sp., and *Jugumella burubaensis* [102,103].

#### 3.2.4. Northeast Gondwanan Unit

This palaeophytogeographical unit includes Northeast Gondwana (Figure 2) and was probably situated as a transition between the tropical-subtropical zones, between 10°–15°south latitude [76]. Similar vegetation is reported from Yunnan (Figure 2—Pd), Xinjiang (Figure 2—P12, Pe) (both China) [50,104], and Vietnam (Figure 2—P13) [105]. 

Rich Ludfordian assemblage flora were described from Victoria, Australia [42,92], situated in the tropical zone of eastern Gondwana (approximately 10° S) [76]. Two stratigraphical levels with plants were recognised, (1) Stratigraphically older *Monograptus* Beds with seven plant taxa (Figure 3—L5), and (2) the Late Centennial Beds with four taxa (Figure 3—L4). Rhyniophytes dominated and zosterophylls were common. The assemblage includes the enigmatic lycopsid *Baragwanathia longifolia* and an undescribed lycophyte-like endemic plant [42,111].

The Yunnan and Xinjiang provinces of China yielded a zosterophyll-dominated flora of Přídolí age (Figure 3—Pd, P12, Pe) with e.g., *Zosterophyllum qujingense* [50]. Dou and Sun [112] and Cai et al. [104] described *Zosterophyllum* sp., *Sciadophyton pristinum*, *Salopella xinjiangensis*, *Cooksonella* (*Junggaria*) *sphaerica*, *Hostinella*, *Psilophytites*, *Parka* and lycophyte-like plants from Xinjinang, north China. Gonez et al. [105] published a plant assemblage from the Van Canh Formation, Vietnam (Figure 3—P13), which may belong to Přídolí, probably *bouceki—transgrendies* biozones and mentioned unbranched axes, cf. *Sporogonites yunnanense*, cf. *Aberlemnia*, cf. *Filiformorama*, and zosterophylls. The locality is close to other Chinese Silurian assemblages situated approximately 20° south latitude [76] on the South China Plate.

### 3.3. Palynological Aspects of Early Land Plants

The combination of the dispersed spore and plant megafossil records is necessary for research into the history of land plants, including the early ones [7,113,114]. Reconstructions of palaeoenvironmental conditions within the Silurian are based mainly on the marine fossil record. Dispersed spores, however, also occurring mainly in near-shore marine sediments, can also be a significant independent factor for successful reconstructions of contemporaneous palaeoenvironments on adjacent palaeocontinents. Indirect palynological evidence of the plant record is available most commonly in the form of spores dispersed in the rock record, and only sometimes in situ, i.e., spores isolated directly from the reproductive organs of plants. The megafossil record of Silurian plants is limited and incomplete compared to that of their spores. Spores have a much higher preservational potential than other plant tissues due to their size and the occurrence of sporopollenin in their exines that effectively protects them against degradation and, thus, leads to better fossilization. There is a very low preservational potential for plants without mineralized mechanical tissues [115,116]. Therefore, the first evidence for land plants is in the form of dispersed spores with a resistant sporopollenin wall [117,118]. Two other palaeobotanically very important aspects of spores are, first, their production in enormous numbers by homosporous plants, and second, their great dispersal potential over long distances by water, but mainly by wind. The optimum size for wind dispersal is 25 μm or less according to Mogensen [119], which corresponds to the diameter of the majority of isospores produced by early land plants (usually 20–40 μm) [120,121].

Cryptospores sensu Stemans [122] appeared earlier than true trilete spores [16]. Their producers/parent plants “cryptosporophytes” [7] are a heterogeneous group of different plant types. It is possible to recognize several types of cryptospores, including enveloped enclosed monads, dyads, and tetrads, naked monads, dyads, and tetrads (Table 1). A large number of dispersed trilete spores in the fossil record reflect the strategy of the earliest plants, which were isosporic, resulting in efficient spreading. Additionally, their abundance in the fossil record significantly exceeds the abundance of the parent plant macrofossils [48].

Based on the dispersed spore record it seems that relatively uniform vegetation was present for 30 million years from the Daipingian to the Llandovery [123].

**Figure 8:**
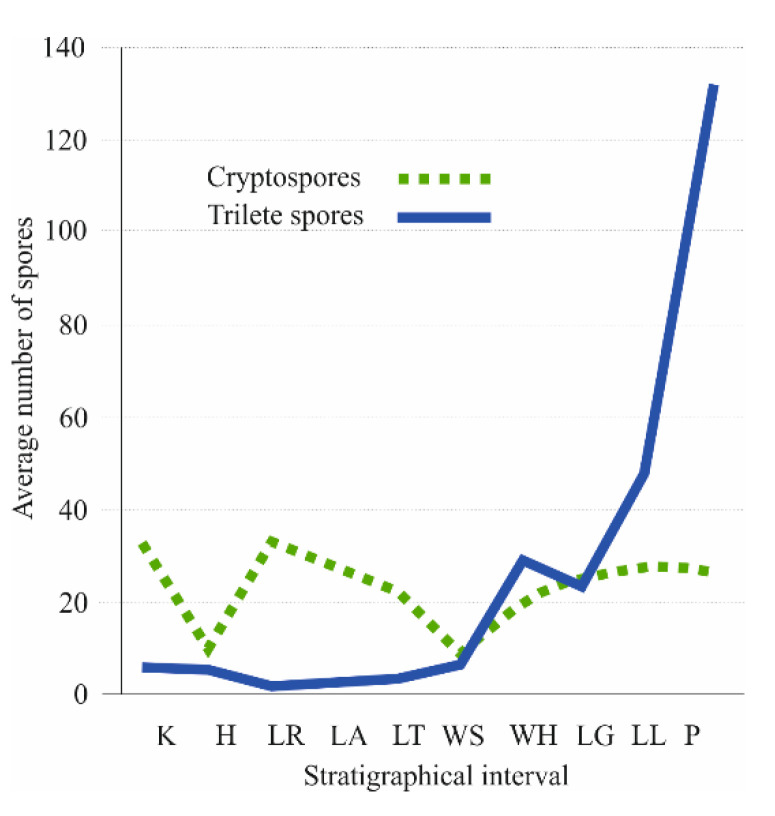
Quantitative curves of cryptospores (dashed line) and trilete spores (full line) from Katian to Přídolí. Horizontal axis represents stratigraphical interval. Abreviations: K—Katian, H—Hirnantian, LR—Llandovery (Rhuddanian), LA—Llandovery (Aeronian), LT—Llandovery (Telychian), WS—lower Wenlock (Sheinwoodian), WH—upper Wenlock (Homerian), LG—lower Ludlow (Gorstian), LL—upper Ludlow (Ludfordian), P—Přídolí. Vertical axis represents average number of trilete spores. Based on data of Wellman et al. [123].

Wellman et al. [123] summarized the records of 787 spore species of all palaeocontinents from Ordovician to Late Silurian. The graphical interpretation of global quantitative data from Katian to Přídolí (Figure 8) shows that biodiversity curves of cryptospores and trilete isospores are different. It also suggests that cryptosporophytes and trilete spore producers had different life strategies and dispersal potential. A prominent cryptospore peak occurs within Rhuddanian (17 genera with 33 species on average), and after a significant decline in the Sheinwoodian (only 9 genera with 9 species) the occurrence from the Homerian to Přídolí is relatively stable (14 genera with 25 species on average). 

The diversity of trilete spores was constant from the Hirnantian to the Sheinwoodian (only 3 genera with 4 species on average); the first global proliferation event of trilete spores, i.e., presumably diversifying ancestors of bryopphytes and tracheophytes [79,123,124] is within Homerian (13 genera with 29 species). After a short period with a decreased number of trilete spore taxa in the Gorstian, a prominent increasing trend is recognized from the Ludfordian (20 genera with 48 species) to the second globally recorded event within the Přídolí (35 genera with 129 species). 

The Homerian event is documented mainly palynologically [79,123] but the Přídolí event is recognized both palynologically and by megafossil plant records [38]. 

Hagström and Mehlqvist [94,114,125] mentioned embryophyte cryptospores of probable Ludfordian Age from approximately 15° south latitude [76] (Figure 2 and Figure 3—L7) from Sweden, i.e., a former eastern part of Laurisia.

All events in the history of Silurian land plants documented here palynologically correspond to changes in the temperature and atmospheric pO_2_ and pCO_2_, as we will discuss.

## 4. Discussion

### 4.1. Response of Silurian Plants to Environmental Perturbations

#### 4.1.1. Wenlock

The Wenlockian flora is known [47] only from the cold temperate zones (Figure 2) of South Laurasia-Northwest Gondwana. It is characterized by a rhyniophytoid-dominated plant assemblage (e.g., Figure 5). Polysporangiophytes of the Wenlock Age are mostly found within the temperate climatic zone to subpolar areas, where significant seasonality can be expected. Generally, the Wenlock is characterized by colder climate culminating in the late Homerian glaciation (Figure 1 and Figure 3). A high diversity of plant types is indicated by the occurrence of dispersed spore assemblages from localities in the Barrandian area (the Prague Basin). For example, the analysis of a Homerian sample coming from a single bed revealed at least ten to twelve types of trilete spore producing plants and three to four types of cryptosporophytes as early as in the Middle Sheinwoodian [78,79]. This fact evoked an idea that relatively rich terrestrial plant assemblages evolved continuously through the Wenlock, through at least two graptolite biozones, in the volcanic islands of the Barrandian area (the Prague Basin), the Czech Republic. Generally, this assemblage (Figure 3—W1 plus Wa) characterized relatively rich vegetation that probably covered physically suitable areas of the Southern hemisphere. Some bryophyte-like microscopic tissue fragments are preserved abundantly within the Homerian (e.g., Figure 6). 

The Homerian glaciation event probably created new large-scale habitats in the physical space that was created by the regression accompanying ice formation. These new habitats were certainly colonized by plants. Plants sensitively responded to seasonal temperature fluctuations and humidity as mentioned by Raymond et al. [47]. Nevertheless, the plants from the temperate zone probably did not have to face the frost during the winter, as suggested by Raymond et al. [47], despite the fact that the Wenlock was a cold period certainly limiting the possibility of plant growth and dispersal (Figure 1). This seems to be supported palynologically because Sheinwoodian cryptospore and trilete spore assemblages reach their minimum diversity here. The first global key proliferation event for trilete spore-producing land plants is documented palynologically after Homerian glaciation (Figure 3). It seems that the Wenlock can be palaeobotanically and palynologically divided into a less environmentally favorable Sheinwoodian interval and a more environmentally suitable late Homerian interval for early land plants.

Edwards and Richardson [46] proposed a ruderal species strategy for rhyniophytoid plants, including a reproductive cycle that was completed during the times of high humidity. Plants persisted as latent spores during periods of unfavorable conditions, although still in a constantly humid environment (mesophytic habitats) [46]. Unfortunately, we do not have evidence for tropical and subtropical plants from Wenlock time.

#### 4.1.2. Ludlow

Since Early Ludlow, the global temperature was increasing until the Middle Ludfordian when this trend was interrupted by a rapid cooling during the Mid-Ludfordian glaciation (Figure 3). After the glaciation, the global temperature reached the same level as before the glaciation. 

Unique Ludlow data come from the equatorial zone where well-preserved plant assemblages from Arctic Canada (Figure 4) and Northern Greenland are reported. This vegetation, characterized by dominance of zosterophylls, has been referred to the North Laurussian palaeophytogeographic unit [47]. Similar plant assemblages of Ludlow age are known from the Northeast Gondwanan (Australia) unit (sensu [47]) where the climate has a transitional character between the tropical and subtropical zones. This area was located approximately 10 ° south latitude during Late Silurian times [76]. Raymond et al. [47] stated that flora from Australia cannot be compared with zosterophyll-dominated vegetation from Bathurst Island of Arctic Canada because different taxa occurred in the two areas, indicating an evolutionary disjunction. 

North Laurussian and northeast Gondwanan units are characterized by the predominance of zosterophylls due to the warm climate (sensu [126]). Zosterophyll-dominated assemblages from northeast Gondwana also yielded a number of rhyniophytes. It is likely that these areas represent the cradle of zosterophyll origin based on their absence in the lower Silurian and on their sudden abundance during Late Silurian in areas where they became the dominant element [50]. So far, we can only speculate why in this warm zone plants appear that have sporangia in the terminal part of their shoots. Certainly, a significant factor was the relatively constant and high temperature and humidity throughout the year, when the growing season was not interrupted by any significant temperature or water stresses. As a basal group of lycophytes, they could have had very similar ecological requirements as the later lycophytes-from the Carboniferous Age. These younger plants preferred a very humid climate and were a significant part of most peat forming assemblages. Based on the absence of zosterophylls in the Early Silurian and the sudden increase in abundance during the Late Silurian in areas where they became the dominant element of the flora, it is likely that these areas also represent the cradle of their origin [50]. In any case it could be assumed that both rhyniophytes and zosterophyllophytes in this period were already equipped with a primitive type of stomata [127]. 

Raymond et al. [47] interpreted assemblages from Australia and Bathurst Island as products of warm zones and mentioned the same ecological conditions accompanying the origin of zosterophyll-like plants. However, it can be concluded that the overall warm climate with episodes of the expansion to higher latitudes (after glacials) of the humid tropical environment during the Silurian undoubtedly led to the evolution of this lycophyte type of plant. Generally, based on palynological results, Gorstian spore and cryptospore assemblages are less numerous and less diverse than previous Homerian associations, which was probably influenced by the preceding Late Homerian glaciation.

#### 4.1.3. The Importance of Mid-Ludfordian Glaciation

The Mid-Ludfordian has been known for one of the largest perturbations in the Phanerozoic carbon cycle, the Mid-Ludfordian Carbon Isotope Excursion, linked with an extinction event recorded in global marine ecosystem. Significant global cooling was recently documented from temperate areas of the Prague Basin and Carnic Alps (peri-Gondwana), as well as from the tropical areas of Baltica (Laurussia) and Australia (Gondwana) based on δ^18^O data [24]. The marked cooling of sea-surface temperatures, coupled with a significant eustatic sea-level fall recorded on all corresponding palaeocontinents, evidences a major glaciation (“Mid-Ludfordian Glaciation”) in polar and subpolar western Gondwana. 

Glaciation passed during Late Ludlow (Ludfordian) accompanied by a significant reduction of average temperature (Figure 1). Large-scale dry land habitat space was created by the Mid-Ludfordian glaciation event and associated sea-level drop, certainly accompanied by plant colonization of these new areas following oceanic regression. We suppose that the vacant ecological windows were occupied by trilete spore producing embryophytes together with bacterial-cyanobacterial mats and still some cryptosporophytes, which colonized coastal areas. Plants established complex and specialized ecosystems on the new land habitats that served as a nutrient source for the rapid global expansion of polysporangiophyte plants, supported also by distinct climatic changes. The expansion is especially apparent for plants preferring warmer and more humid conditions. Raymond et al. [47] supposed that the lycophyte genus *Baragwanathia* is typical for warm and humid climatic zones. However, *Baragwanathia brevifolia* and another basal lycophyte, *Aberlemnia bohemica,* are described also from a higher latitude of the peri-Gondwana Realm (namely Barrandian area, the Prague Basin, Czech Republic) and a higher stratigraphical level (Přídolí) [38,43]. It seems that the expansion of *Baragwanathia*-like plants to higher latitudes is associated with increased humidity during glaciation and following global warming after the Mid-Ludfordian glacial. 

A similar shift to higher latitudes of lycophytes and zosterophylls can be traced in other localities that belong to the temperate zone of the South Laurussian-Northwest Gondwanan unit [47]. For example, a Podolian assemblage (Figure 2—P8) from eastern Laurussia shows dominance of rhyniophytoids, including *Cooksonia pertoni*, *C. hemisphaerica*, and mainly *Zosterophyllum* sp. and *Lycopodolica,* rather typical for the warmer equatorial Silurian zones. The Ludfortian is palynologically characterized by the increasing variability of spore and cryptospore assemblages with a high number of new taxa.

#### 4.1.4. Přídolí

The prominent rise in temperature during the Přídolí [128] was permissive of the evolution of new forms of plants. Transgression contributed to the frequent transport of land plants into the sea and created Silurian taphonomic windows.

The number of Silurian localities with rhyniophytoid-dominated vegetation (including the typical occurrence of *Cooksonia*) is higher during the Přídolí than during the Ludlow. This homogenization was based by Raymond et al. [47] on floras of similar composition in both areas where the rhyniophytoid-dominated flora is present and includes some common taxa, e.g., *Cooksonia hemisphaerica*. In this context however, the occurrence of *C. hemisphaerica s.s.* in Northwest Gondwana is still questionable and needs re-evaluation. A similar situation is also found with respect to a comparison of the character of assemblages from Great Britain and Bolivia [47]. Raymond et al. [47] explained this similarity by the seasonality that equally affected plants from SE Laurussia in the subtropical zone and Northwest Gondwana or peri-Gondwana in the temperate zone. 

The general plant character from China and Vietnam shows some similarities [105,126] to Ludlowian vegetation from tropical Bathurst Island or maybe subtropical Victoria, Australia, in having *Zosterophyllum*-like plants as dominant. Plants of Přídolí age are known from the Kazakhstanian unit situated in the subtropical zone of the North hemisphere. Northern latitude floras differ from those of environmentally similar southern latitudes in the common occurrence of *Zosterophyllum*-like plants in the former. Edwards and Wellman [45] pointed out this phenomenon and placed the Kazakhstanian flora on the South China Block (Xinjiang flora). Relatively rich localities already appear in several areas during the Přídolí and this trend continues across the Silurian/Devonian boundary into the Early Devonian. The number and variations of spore and cryptospore assemblages rapidly increased during the Přídolí Series, reflecting the second globally important key event for land plants.

## 5. Conclusions

Atmospheric oxygen steadily rose from the Middle Ordovician and through almost the entire Silurian. By the beginning of the Wenlock, oxygen levels surpassed the current level for the first time in the Earth’s history, and at the end of Silurian attained 35% [64,129]. This trend supports previous conclusions [49,64] and our own herein (based on our data and compiled fossil data as discussed) that, despite climate fluctuations, terrestrial flora expanded during the Silurian. We hope we have relaxed at least a small bit of constraints on Silurian phytogeography and support the conclusions of Edwards [49]. Current phylo-genomic/-stratigraphy dating of the embryophyte split into tracheophytes and bryophytes ranges from approximately 440 up to 800 Myr for tracheophytes [15,18]. This on one hand indicates uncertainty related to the timing of this major evolutionary cross-roads in land plant evolution. On the other hand, it also indicates that the tracheophyte lineage was certainly well established in the Silurian. Our analysis of spore diversity evolution clearly indicates a very distinct exponential evolutionary acceleration of trilete spore producers starting in the lower Wenlock (and continuing in fact until today—Figure 8). This expansion of trilete spore producers is also reflected in an apparent geographical spread of documented macrofossils between the Wenlock and Ludlow/Přídolí (Figure 2). Cryptospore-producers, which dominated Silurian flora until the upper Llandovery stagnated evolutionarily until their Devonian extinction. In a more detailed attempt to correlate trilete spore diversity and global temperature changes (Figure 3), we conclude that after three distinct Middle to Late Silurian glaciations we can clearly see an increase of diversity correlated with the rise of the global temperature after the glaciations ended. Interestingly, while Homerian glaciation did not affect the diversity of cryptospores, it resulted in the distinct decline in the diversity of trilete spores. This indicates that there was a different sensitivity of cryptosporophytes vs. trilete spores-producers to the global cold temperature. In respect to fragmentary Silurian phytogeography data summarized vividly by Edwards [49] (see above), our data essentially support her conclusions that the four distinct plant assemblages correlate very well with the four palaeocontinents. 

It certainly is very difficult even to speculate about the physiological character of Silurian plants. However, both detailed, enhanced anatomical knowledge of the immediately following Early Devonian rhyniophyte flora, and also advancements in phylogenomic and phylostratigraphic analyses provide important facts allowing an informed reconstruction of at least some aspects of the biology of Silurian trilete spore-producing plants (we do not speculate here about the biology of cryptosporophytes.) Based on all the current available fossil data, the Silurian flora is composed of different types of polysporangiate plants (including cryptophytes)—any reliable evidence for the monosporangiates is absent. There are no identified gametophytes of any Silurian plant. Thus, the first reliable life cycles/alteration of gametophyte and sporophyte generations is not known before that of several representatives of Devonian Rhynie chert flora [130]. In relation to plant physiology, stomata are one of the crucial ecophysiological features related to both temperature, water availability and atmospheric composition. Based on both phylogenomics-indicating reduction or loss of stomatal functioning in the common ancestor of extant embryophytes in bryophytes [127], as well as the palaeobotanical demonstration of distinct stomata described both on sporophytes and gametophytes of Rhynie chert plants [131], we should accept that the common ancestor of both mosses and tracheophytes had stomata—most probably already dynamically regulated (opening-closing) by the abscisic acid signalling pathway [127,132]. This pathway is crucial for the optimization of land-plant water economy and survival of water stresses both during day-night cycles, but importantly also during seasons allowing local adaptations dependent on latitude. As is the case for abscisic acid signalling, the evolution of lignified secondary-thickened conductive tissues is well documented in Rhynie chert plants. Lignification most probably started much earlier and precursors of tracheophyte xylem and phloem were with high probability also present in the common Bryophyta-Tracheophyta ancestor. As in the case of stomata [127], it is possible that secondary simplification accompanied the evolution of other traits in the Bryophyta. Major transcriptional regulators of xylem development are also encoded in bryophyte genomes and VND-NST-SOMBRERO (VNS) genes—vessel-element differentiation master regulators—from *Sphagnum* moss (which does not have hydroids and instead uses hyaline cells with thickened, helical-patterned cell walls and pores to store water in the leaves) and induces secondary cell wall thickening when ectopically expressed in the angiosperm *Nicotiana benthamiana* [133]. The anatomy of Silurian (and Devonian) plant fossils/assemblages, along with recent phylogenomic analyses, clearly indicate highly terrestrialized advanced plant types with likely a great ability to resist different dry land stress conditions and to evolve on different palaeocontinets into quite disjunctive specific floras/plant assemblages certainly also in response to different geological, geographical and climatic conditions.

## Figures and Tables

**Table 1:** Quantitative occurrence of main types of cryptospores, hilate spores and true trilete spores from Llandovery to Přídolí.

Age	Envelope Enclosed Monads	Envelope Enclosed Dyads	Envelope Enclosed Tetrad	Naked Monads	Naked Dyads	Naked Tetrads	Hilate Spores	Trilete Spores
Přídolí	█ █	█ █	█ █	██ ██	█ █	█ █	███ ███	███ ███
Ludlow	█ █	█ █	█ █	██ ██	█ █	█ █	██ ██	██ ██
Wenlock	█ █	█ █	█ █	█ █	██ ██	██ ██	██ ██	██ ██
Llandovery	█ █	█ █	█ █	█ █	██ ██	██ ██	█ █	█ █
	██ ██	███ ███	███ ███	█ █	███ ███	███ ███	█ █	█ █

## Data Availability

The data presented in this study are available on request from the corresponding author.
